# Supplementary material for: Diffusion MRI with double diffusion encoding and variable mixing times disentangles water exchange from transient kurtosis
Source: Sci Rep. 2025 Mar 13;15:8747. doi: 10.1038/s41598-025-93084-4 (PMC11906880; doi:10.1038/s41598-025-93084-4)
Supplement: Supplementary file 1 — Supplementary Information. [file 41598_2025_93084_MOESM1_ESM.pdf]

7 Appendix

7.1 On the relation between MGE and filter-exchange imaging

A relation between MGE and FEXI can be obtained by deriving the dependence of the ADC on the mixing time, defined according to

$$D'(t_m) = -\frac{1}{b_d}\left[\ln E_{f+d} - \ln E_f\right] \qquad (A.1)$$

where E_{f+d} and E_d are the diffusion-weighted signals from acquisitions with the filtering and detection block active and detection only, respectively. These signals can be predicted using the MGE framework. We assume DDE acquisitions with short pulses which allows the exchange-sensitised square of the b-tensor to be written

$$\mathbf{B}^{\otimes 2}(k) = 2\int_0^T \mathbb{Q}_4(t)\exp(-kt)\,\mathrm{d}t = \mathbb{Q}_4^0\,\Delta h_0(k,\Delta) + 2\mathbb{Q}_4^1\,\Delta h_1(k,\Delta,t_m) \qquad (A.2)$$

where $\mathbb{Q}_4^0 = \mathbb{Q}_4(0)$ and $\mathbb{Q}_4^1 = \mathbb{Q}_4(t_m+\Delta)$, $h_0(k,\Delta) = \frac{\Delta k + e^{-\Delta k} - 1}{\Delta^2 k^2}$ and
$h_1(k,\Delta,t_m) = \left[\frac{e^{-t_m k} - e^{-k(\Delta+t_m)} - \Delta k e^{-k(\Delta+t_m)}}{\Delta^2 k^2}\right] + \left[\frac{e^{-kT} + kTe^{-k(\Delta+t_m)} - e^{-k(\Delta+t_m)} - k\Delta e^{-k(\Delta+t_m)} - kt_m e^{-k(\Delta+t_m)}}{\Delta^2 k^2}\right]$.

That is

$$h_1(k,\Delta,t_m) = \frac{e^{-t_m k} - 2e^{-k(\Delta+t_m)} + e^{-k(2\Delta+t_m)}}{\Delta^2 k^2} \qquad (A.3)$$

which is the second term of Eq. 16 in the main text.

Assuming $k\Delta \ll 1$ gives $h_0(k,\Delta) \approx 1/2$ and $h_1(k,\Delta,t_m) \approx e^{-kt_m}$. $\mathbf{B}^{\otimes 2}(k)$ can then be written

$$\mathbf{B}^{\otimes 2}(k) = \mathbf{B}_f^{\otimes 2} + \mathbf{B}_d^{\otimes 2} + 2\mathbf{B}_f \otimes \mathbf{B}_d e^{-kt_m} \qquad (A.4)$$

The isotropic and anisotropic projections of $\mathbf{B}^{\otimes 2}(k)$ can then be expressed

$$b_I^2(k) = \left(b_f^2 + b_d^2\right) + 2b_f b_d e^{-kt_m} \qquad (A.5)$$

$$b_A^2(k) = \left(b_f^2 + b_d^2\right) + 2b'_\Delta b_f b_d e^{-kt_m} \qquad (A.6)$$

where $b'_\Delta = 1$ for SDE and parallel DDE and $b'_\Delta = -\frac{1}{2}$ for orthogonal DDE. For simplicity, we re-write the MGE representation as:

$$\ln E \approx -bD + \frac{1}{2}[b_I^2(k)V_I + b_\Delta^2(k)V_A] \qquad (A.7)$$

Now the desired signal difference is given by

$$D'(t_m) = \langle D\rangle - \frac{1}{2}b_d[V_I + V_A] - \frac{1}{2}b_f e^{-kt_m}[V_I + b'_\Delta V_A] \qquad (A.8)$$

At long mixing times, $t_m \to \infty$, the diffusivity approaches its equilibrium value

$$D^{eq} = \langle D\rangle - \frac{1}{2}b_d[V_I + V_A] \approx \langle D\rangle \qquad (A.9)$$

where the approximation assumes b_d is low. Defining the filter efficiency as

$$\sigma = 2b_\mathrm{f}\frac{(V_I + b'_\Delta V_A)}{\langle D\rangle} \qquad (A.10)$$

gives us the well-known FEXI signal equation:

$$D'(t_\mathrm{m}) = D^\mathrm{eq}[1 - \sigma e^{-kt_m}]. \qquad (A.11)$$

Note that Eq. A.10 implies that the filter efficiency for orthogonal DDE can be negative if $V_A > 2 \cdot V_I$.

7.2 On the relation between tMGE and SMEX/NEXI

It is important to highlight the link between tMGE and the contemporary SMEX/NEXI [1–3] models for mapping exchange with diffusion MRI. These models are based on two exchanging Gaussian components, where we expect zero transient kurtosis but non-zero microscopic kurtosis. For SMEX/NEXI, the signal is given generally by

$$\ln S(b_1, b_2, \Delta, t_m, \cos\psi) \approx -(b_1 + b_2)\,\bar{D} + \frac{1}{6}(b_1^2 + b_2^2)\bar{D}^2 K + b_1 b_2\left(\cos^2\psi\,(Z_{3333} - Z_{1133}) + Z_{1133}\right) \qquad (A.12)$$

where

$$\bar{D} = f_n\left(\frac{D_n}{3} - D_e\right) + D_e \qquad (A.13)$$

$$K = K_\infty + K_0\,\frac{k\Delta - (1 - e^{-k\Delta})}{k^2\Delta^2} \qquad (A.14)$$

$$Z_{3333} = \frac{\bar{D}^2}{6}\left(e^{-kt_m}\,\frac{(1 - e^{-k\Delta})^2}{k^2\Delta^2} K_0 + 2K_\infty\right) \qquad (A.15)$$

$$Z_{1133} = \frac{\bar{D}^2}{6}\left(e^{-kt_m}\,\frac{(1 - e^{-k\Delta})^2}{k^2\Delta^2}\left(K_0 - 3\,\frac{1 - f_n}{f_n} K_\infty\right) - K_\infty\right) \qquad (A.16)$$

and

$$K_0 = \frac{2}{5}\,\frac{f_n(1 - f_n)}{\bar{D}^2}\,(3D_n^2 - 10D_nD_e + 15D_e^2) \qquad (A.17)$$

$$K_\infty = \frac{4}{15}\,\frac{f_n^2 D_n^2}{\bar{D}^2} \qquad (A.18)$$

Subscripts “n” and “e” represent “neurite” and “extracellular”, respectively. The SDE signal under this representation is given by

$$\ln S_{SDE}(b, \Delta) \approx -b\,\bar{D} + \frac{1}{6} b^2 \bar{D}^2\left(\frac{1}{2} K_0 h_{SDE}(k, \Delta) + K_\infty\right) \qquad (A.19)$$

Where

$$h_{SDE}(k, \Delta) = 2\,\frac{k\Delta - (1 - e^{-k\Delta})}{k^2\Delta^2} \qquad (A.20)$$

as defined in the main text. The parallel DDE signal is given by

$$\ln S_{DDE}(b_1, b_2, \Delta, t_m, 1) \approx -(b_1 + b_2)\bar{D} + \frac{1}{6}(b_1^2 + b_2^2)\bar{D}^2 K + b_1 b_2 Z_{3333}$$

$$= -(b_1 + b_2)\overline{D} + \frac{1}{6}(b_1^2 + b_2^2)\overline{D}^2\left(\frac{1}{2}K_0 h_{SDE}(k,\Delta) + K_\infty\right)$$

$$+ b_1 b_2 \frac{\overline{D}^2}{6}\left(e^{-kt_m}\frac{1 - 2e^{-k\Delta} + e^{-2k\Delta}}{k^2\Delta^2}K_0 + 2K_\infty\right)$$

$$= -(b_1 + b_2)\overline{D} + \frac{1}{6}(b_1^2 + b_2^2)\overline{D}^2\left(\frac{1}{2}K_0 h_{SDE}(k,\Delta) + K_\infty\right)$$

$$+ b_1 b_2 \frac{\overline{D}^2}{6}\left(\frac{e^{-kt_m} - 2e^{-k(\Delta+t_m)} + e^{-k(2\Delta+t_m)}}{k^2\Delta^2}K_0 + 2K_\infty\right)$$

$$= -(b_1 + b_2)\overline{D}$$

$$+ \frac{1}{6}\overline{D}^2 K_0\left(\frac{1}{2}(b_1^2 + b_2^2)h_{SDE}(k,\Delta) + b_1 b_2 \frac{e^{-kt_m} - 2e^{-k(\Delta+t_m)} + e^{-k(2\Delta+t_m)}}{k^2\Delta^2}\right)$$

$$+ \frac{1}{6}\overline{D}^2 K_\infty (b_1^2 + b_2^2 + 2b_1 b_2) \qquad (A.21)$$

The long mixing time condition of CTI is obtained by letting $t_m \to \infty$ which gives

$$\ln S_{DDE}(b_1, b_2, \Delta, t_m \to \infty, 1) \approx -(b_1 + b_2)\overline{D} + \frac{1}{6}\overline{D}^2 K_0\left(\frac{1}{2}(b_1^2 + b_2^2)h_{SDE}(k,\Delta)\right)$$

$$+ \frac{1}{6}(b_1 + b_2)^2\overline{D}^2 K_\infty \qquad (A.22)$$

Microscopic kurtosis can be computed from the SDE-DDE signal difference. We set, for simplicity, $b_1 = b_2 = \frac{1}{2}b$ such that

$$\ln S_{DDE}(b, \Delta, t_m \to \infty, 1) \approx -b\overline{D} + \frac{1}{6}\overline{D}^2 b^2\left(K_0 \frac{1}{4}h_{SDE}(k,\Delta) + K_\infty\right) \qquad (A.23)$$

Microscopic kurtosis is now given by

$$K_\mu = \frac{12}{\overline{D}^2 b^2}\left(\ln S_{SDE}(b,\Delta) - \ln S_{DDE}(b,\Delta,t_m \to \infty, 1)\right)$$

$$= \frac{12}{\overline{D}^2 b^2}\left(\frac{1}{24}\overline{D}^2 b^2 K_0 h_{SDE}(k,\Delta)\right)$$

$$= \frac{1}{2}K_0 h_{SDE}(k,\Delta) \qquad (A.24)$$

which agrees with Eq. 21 in the main text. In summary, in the isotropic two-Gaussian-component system treated above, there is no transient kurtosis and the effect of exchange is a temporal decline of the intercompartmental kurtosis. This decline is captured by microscopic kurtosis in CTI and by k in tMGE.

It is also worth comparing the exchange-weighting properties of orthogonal DDE acquisitions as described by the SMEX/NEXI above versus tMGE. For orthogonal DDE, Eq. A.12 gives

$$\ln S_{DDE}(b_1, b_2, \Delta, t_m, 0) \approx -(b_1 + b_2)\overline{D} + \frac{1}{6}(b_1^2 + b_2^2)\overline{D}^2 K + b_1 b_2 Z_{1133}$$

$$= -(b_1 + b_2)\overline{D} + \frac{1}{6}(b_1^2 + b_2^2)\overline{D}^2 K$$
$$+ b_1 b_2 \frac{\overline{D}^2}{6}\left(e^{-kt_m}\frac{(1 - e^{-k\Delta})^2}{k^2\Delta^2}\left(K_0 - 3\frac{1 - f_n}{f_n}K_\infty\right) - K_\infty\right)$$

$$= -(b_1 + b_2)\overline{D} + \frac{1}{6}(b_1^2 + b_2^2)\overline{D}^2\left(\frac{1}{2}K_0 h_{SDE}(k, \Delta) + K_\infty\right)$$
$$+ b_1 b_2 \frac{\overline{D}^2}{6}\left(f_{DDE}(k, \Delta, t_m)\left(K_0 - 3\frac{1 - f_n}{f_n}K_\infty\right) - K_\infty\right) \qquad (A.25)$$

where

$$f_{DDE}(k, \Delta, t_m) = e^{-kt_m}\frac{(1 - e^{-k\Delta})^2}{k^2\Delta^2} \qquad (A.26)$$

Eq. A.25 can be further simplified to

$$\ln S_{DDE}(b_1, b_2, \Delta, t_m, 0) \approx -(b_1 + b_2)\overline{D} + \frac{1}{6}(b_1^2 + b_2^2)\overline{D}^2\left(\frac{1}{2}K_0 h_{SDE}(k, \Delta) + K_\infty\right)$$
$$+ b_1 b_2 \frac{\overline{D}^2}{6}\left(f_{DDE}(k, \Delta, t_m)\left(K_0 - 3\frac{1 - f_n}{f_n}K_\infty\right) - K_\infty\right) \qquad (A.27)$$

To simplify the comparison with tMGE, we set $b_1 = b_2 = \frac{1}{2}b$, which yields

$$\ln S_{DDE}(b_1, b_2, \Delta, t_m, 0)$$
$$\approx -b\overline{D} + \frac{1}{24}b^2\overline{D}^2(K_0 h_{SDE}(k, \Delta) + 2K_\infty)$$
$$+ \frac{1}{24}b^2\overline{D}^2\left(f_{DDE}(k, \Delta, t_m)\left(K_0 - 3\frac{1 - f_n}{f_n}K_\infty\right) - K_\infty\right)$$

$$= -b\overline{D} + \frac{1}{24}b^2\overline{D}^2(K_0(h_{SDE}(k, \Delta) + f_{DDE}(k, \Delta, t_m)) + 2K_\infty)$$
$$+ \frac{1}{24}b^2\overline{D}^2\left(f_{DDE}(k, \Delta, t_m)\left(-3\frac{1 - f_n}{f_n}K_\infty\right) - K_\infty\right)$$

$$= -b\overline{D} + \frac{1}{24}b^2\overline{D}^2 h_{SDE}(k, \Delta)K_0 + \frac{1}{24}b^2\overline{D}^2 f_{DDE}(k, \Delta, t_m)\left(K_0 - 3\frac{1 - f_n}{f_n}K_\infty\right)$$
$$+ \frac{1}{24}b^2\overline{D}^2 K_\infty \qquad (A.28)$$

According to tMGE (Eq. 36 in the main text),

$$\ln E \approx -bD + \frac{1}{6}b^2D^2h(k)[K_I^0 + h_\Delta^2(k)K_A^0] + \frac{1}{6}b^2D^2\left[K_I^\infty + b_\Delta^2K_A^\infty + b_\mu^2K_{tr}\right] \qquad (A.29)$$

We neglect the transient kurtosis term, and note that, according to the discussion in Section 7.1 above, the projections of the exchange-encoding tensor have the explicit forms

$$h(k) = (b_1^2 + b_2^2)h_{SDE}(k, \Delta) + 2b_1b_2f_{DDE}(k, \Delta, t_m) \qquad (A.30)$$

and

$$h(k)h_\Delta^2(k) = (b_1^2 + b_2^2)h_{SDE}(k, \Delta) - b_1b_2f_{DDE}(k, \Delta, t_m) \qquad (A.31)$$

Rewriting Eq. A.29 with these expressions provides

$$\ln S_{DDE}(b_1, b_2, \Delta, t_m, 0) \approx -(b_1 + b_2)\overline{D} + \tfrac{1}{6}(b_1^2 + b_2^2)\overline{D}^2\left(K_I^0h_{SDE}(k, \Delta) + K_A^0h_{SDE}(k, \Delta)\right) + \tfrac{1}{6}b_1b_2\overline{D}^2(2K_I^0f_{DDE}(k, \Delta, t_m) - K_A^0f_{DDE}(k, \Delta, t_m)) + \tfrac{1}{6}(b_1 + b_2)^2\overline{D}^2\left(K_I^\infty + \tfrac{1}{4}K_A^\infty\right) \qquad (A.32)$$

Setting $b_1 = b_2 = \frac{1}{2}b$ gives

$$\begin{aligned}\ln S_{DDE}(b_1, b_2, \Delta, t_m, 0) &\approx -b\overline{D} + \frac{1}{12}b^2\overline{D}^2\left(K_I^0h_{SDE}(k, \Delta) + K_A^0h_{SDE}(k, \Delta)\right) \\ &\quad + \frac{1}{24}b^2\overline{D}^2(2K_I^0f_{DDE}(k, \Delta, t_m) - K_A^0f_{DDE}(k, \Delta, t_m)) + \frac{1}{6}b^2\overline{D}^2\left(K_I^\infty + \frac{1}{4}K_A^\infty\right) \\ &= -b\overline{D} + \frac{1}{24}b^2\overline{D}^2h_{SDE}(k, \Delta)(K_I^0 + K_A^0) + \frac{1}{24}b^2\overline{D}^2f_{DDE}(k, \Delta, t_m)(2K_I^0 - K_A^0) \\ &\quad + \frac{1}{24}b^2\overline{D}^2(4K_I^\infty + K_A^\infty) \qquad (A.33)\end{aligned}$$

Comparing Eq. A.28 and A.33, we see that SMEX/NEXI and tMGE predict the same orthogonal DDE signals under the conditions

$$K_0 = K_I^0 + K_A^0 \qquad (A.34)$$

$$2K_I^0 - K_A^0 = K_0 - 3\frac{1 - f_n}{f_n}K_\infty \qquad (A.35)$$

$$K_\infty = 4K_I^\infty + K_A^\infty \qquad (A.36)$$

References

1. Ghazi, N., Coelho, S., Shemesh, N. & Jespersen, S. N. Tensor encoded diffusion weighting improves model parameter estimation of SMEX/NEXI. *Proc. ISMRM Singap.* (2024).
2. Olesen, J. L., Østergaard, L., Shemesh, N. & Jespersen, S. N. Diffusion time dependence, power-law scaling, and exchange in gray matter. *NeuroImage* **251**, 118976 (2022).
3. Jespersen, S. N. Equivalence of double and single wave vector diffusion contrast at low diffusion weighting. *NMR Biomed.* **25**, 813–818 (2012).

7.3 Supplementary figures

This section contains CTI kurtosis estimates in substrates of spheres and beads, where the intracellular and extracellular signals were analysed separately. MGE kurtosis and exchange estimates in multi-Gaussian components at slow exchange and in spheres that feature non-Gaussian diffusion with exchange are also presented herein. Finally, CTI and tMGE are compared in the substrate of parallel cylinders.

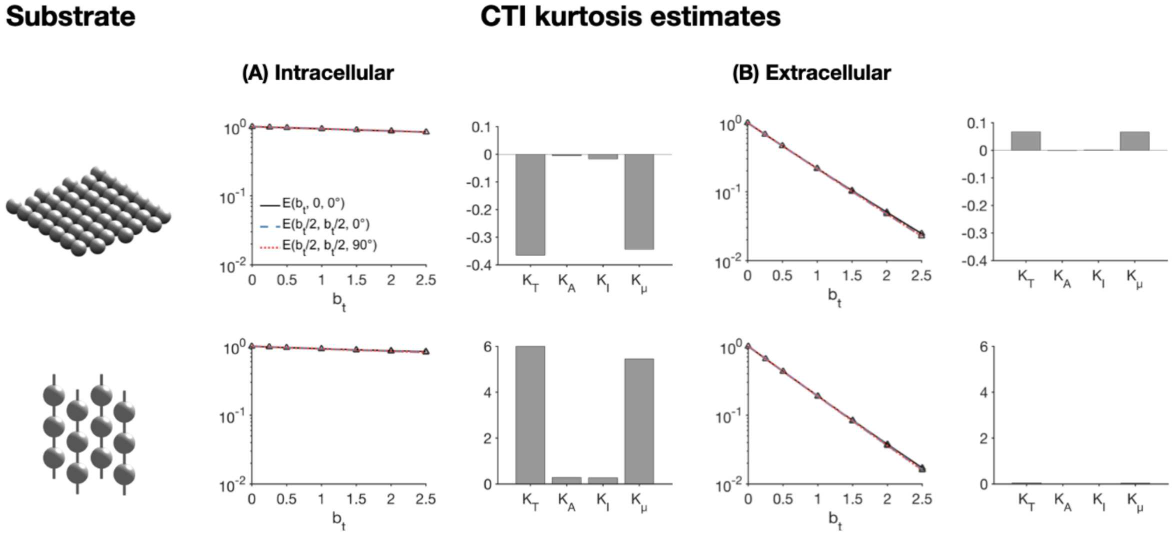

Figure A1: CTI parameter estimates in the substrates of 6 μm-diameter spheres and beads. The estimates were obtained by separately fitting the CTI signal representation to the intracellular and extracellular signals. For cylinders, the intracellular signals give a large negative microscopic kurtosis, while the extracellular space has a small positive microscopic kurtosis. For beads, there is a large positive microscopic kurtosis from the intracellular signals and a very small positive microscopic kurtosis from the extracellular signals.

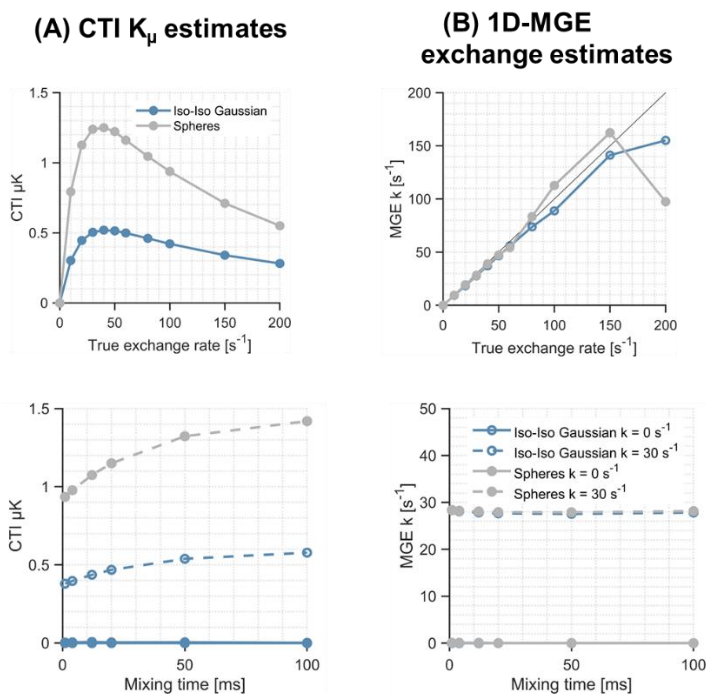

Figure A2: Variation of CTI and 1D-MGE parameter estimates with exchange rate and mixing time. Results are shown in substrates of regular spheres in exchange with the extracellular space and isotropic Gaussian components in exchange. This figure is the equivalent of Fig. 5 in the main text but generated with the clinical protocol.

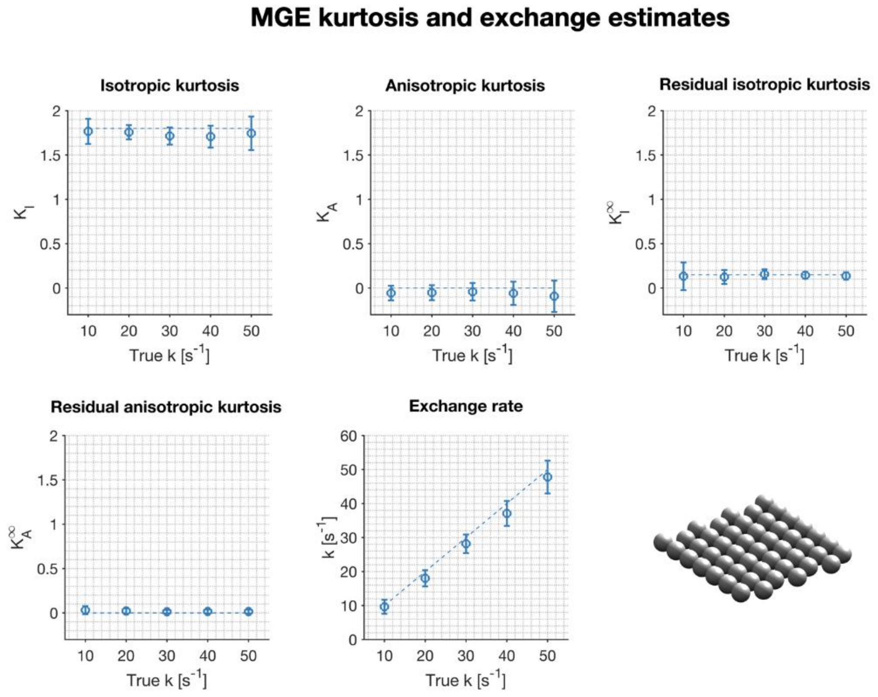

Figure A3: MGE parameter estimates in spheres at SNR = 200. The dashed lines indicate ground truth. The kurtosis is dominated by the isotropic components as expected. All estimates show good agreement with ground truth, with a small bias that can be explained by higher-order effects associated with the cumulant expansion. The error bias represent 1 standard deviation.

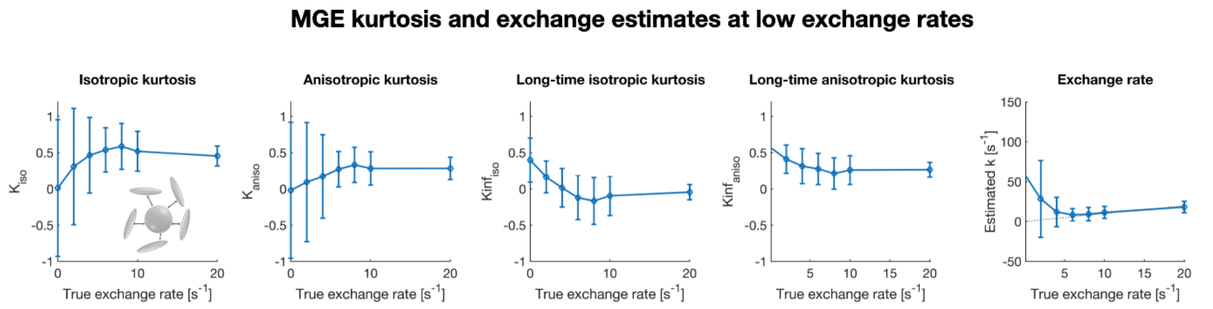

Figure A4: MGE parameter estimates for anisotropic Gaussian components in exchange with an isotropic component. This is the equivalent of Fig. 4 but for lower exchange rates. The dependence on exchange rate of the kurtosis estimates, the bias in estimated exchange rates and the relatively large uncertainties at low exchange rates indicate the difficulty of inverting the MGE signal representation around k = 0.

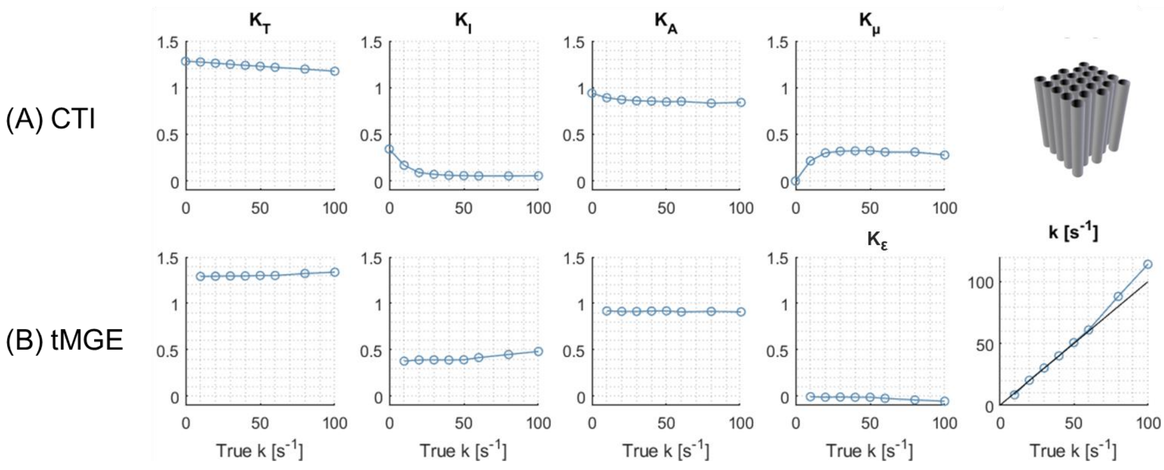

Figure A5: Evaluation of tMGE in a substrate of parallel cylinders. Fitting tMGE allows disentanglement of two sources of microscopic kurtosis: exchange and transient kurtosis.

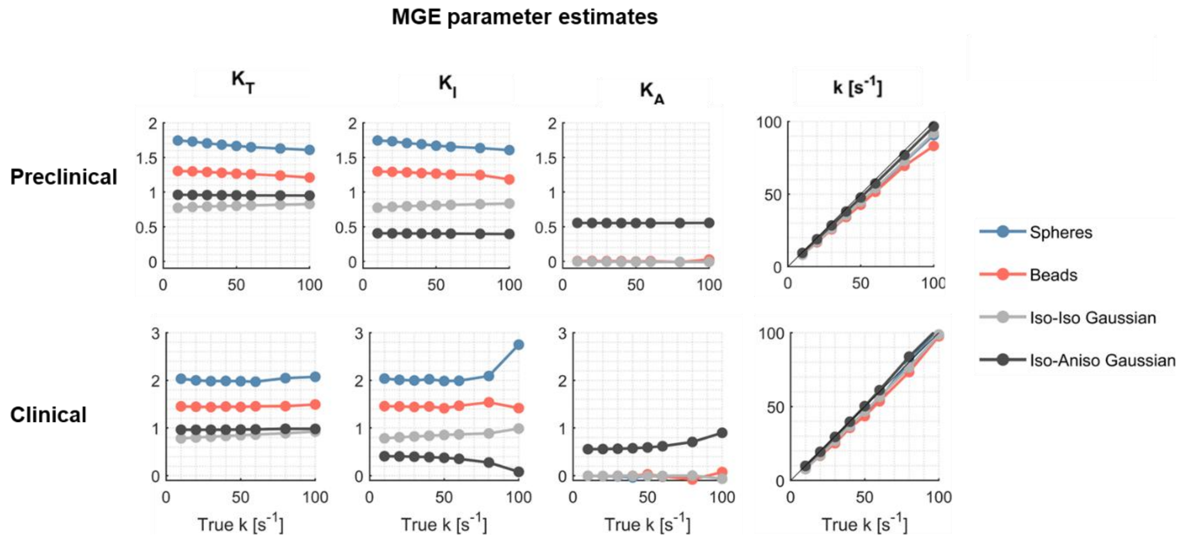

Figure A6: MGE parameter estimates in different substrates with varying rates of exchange. With the preclinical protocol, all kurtosis estimates are largely independent of the underlying exchange rate, and the exchange estimates agree well with the ground truth. Similar trends are observed with the clinical protocol, with the exception of spheres and anisotropic Gaussian where there is a bias in isotropic and anisotropic kurtosis estimates at the fastest exchange rate simulated.
